# Investigation of Deoxynivalenol Contamination in Local Area and Evaluation of Its Multiple Intestinal Toxicity

**DOI:** 10.3390/toxins16080353

**Published:** 2024-08-12

**Authors:** Yebo Wang, Minjie Zhang, Ke Li, Chune Zhang, Honglei Tian, Ying Luo

**Affiliations:** 1 College of Food Engineering and Nutritional Science, Shaanxi Normal University, Xi’an 710119, China; wangyebo0525@163.com (Y.W.); 2023301355@snnu.edu.cn (M.Z.); li_ke@snnu.edu.cn (K.L.); 2 Ningxia Hui Autonomous Region Grain and Oil Product Quality Inspection Center, Yinchuan 750001, China; zhchune@163.com

**Keywords:** deoxynivalenol, contamination, intestinal toxicity, gut microbiota, transcription analysis

## Abstract

Deoxynivalenol (DON) is a mycotoxin produced by *Fusarium fungi* widespread in wheat, corn, barley and other grain crops, posing the potential for being toxic to human and animal health, especially in the small intestine, which is the primary target organ for defense against the invasion of toxins. This study firstly investigated DON contamination in a local area of a wheat production district in China. Subsequently, the mechanism of DON toxicity was analyzed through cellular molecular biology combining with intestinal flora and gene transcription analysis; the results indicated that DON exposure can decrease IPEC−J2 cell viability and antioxidant capacity, stimulate the secretion and expression of proinflammatory factors, destroy the gut microbiota and affect normal functions of the body. It is illustrated that DON could induce intestinal damage through structural damage, functional injury and even intestinal internal environment disturbance, and, also, these intestinal toxicity effects are intrinsically interrelated. This study may provide multifaceted information for the treatment of intestinal injury induced by DON.

## 1. Introduction

Deoxynivalenol (DON), primarily produced by *Fusarium graminearum* and *Fusarium culmorum*, is a common mycotoxin pollutant in grain and grain-based products (wheat, corn, barley, etc.) [1,2]. The DON contamination rate once ranked first among all kinds of mycotoxins according to public statistics; grains contaminated with DON can firstly lead to a decline in quality and, more importantly, the human body can be exposed to DON directly through consumption of contaminated grain products and/or indirectly through consumption of animal products (meat products, milk, eggs, etc.) that have been compromised by DON [3,4]. A high DON contamination rate and its toxicity have caused it to become a serious threat to human health; the functions of the nervous, immune, reproductive and intestinal systems can be impaired and clinical symptoms include nausea, vomiting, abdominal pain, diarrhea, headache and fever after DON exposure [5,6].

In recent years, with the increase in extreme weather such as global warming and greenhouse gas emissions, the pollution trend of DON has increased, and higher concentrations of DON have been detected in samples from temperate regions of North America, Northern Europe, Central Europe and East Asia [7]. Climatic conditions are one of the serious causes of DON pollution. A survey of 23 counties in China’s rainy regions along the Yangtze River showed that 1192 out of 1846 isolates produced DON according to a survey of wheat samples harvested in China’s Shaanxi, Ningxia, Gansu and Xinjiang regions. A total of 82% of the samples were positive for DON, and 10% of the samples were above the maximum limit of 1000 µg/kg [8,9].

Susceptibility to DON varies greatly among animals, and pigs and humans are highly sensitive to DON with similar absorption rates, high bioavailability and long clearance times. This is due to the lack of detoxification microorganisms for DON in these two animals, which mainly rely on liver glycosylation and urine excretion [10]. The target organs are attacked by DON mainly through inflammation, oxidative stress and apoptosis. DON can cause severe liver damage through hepatocellular oxidative stress and decrease the antioxidant capacity in the body [11,12]. After DON exposure, liver weight can decrease and a disordered arrangement of hepatocytes along with noticeable hepatocellular siltation and inflammatory infiltration could be found [12]. In addition, after being treated with DON, significant apoptosis was observed in hepatocytes, ROS levels increased and the levels of antioxidant-related transcripts, including GSTO1, GSTA1, HMOX2, GPX4, SOD1 and CAT, were all decreased [11]. Furthermore, some studies have shown that exposure to DON also caused damage to other target organs such as the kidney and spleen. The weight of the kidney and spleen exhibited an increase after DON exposure, which is a sign of organ edema [13]. DON exposure can disrupt energy metabolism in the kidney by reducing the levels of ribitol, glycerol 1−phosphate and other compounds; meanwhile, it can also lead to dysfunction in immune function and nucleotide metabolism in the spleen [14]. 

Among the attacked organs, the intestine is the most vulnerable and most damaged target because it is not only the key organ for absorbing nutrients but also the first barrier against the invasion of various toxins [15]. Exposure to DON may lead to the destruction of the intestinal wall morphology, villus height and the number of goblet cells and lymphocytes [4,16,17]. According to Song (2022) [4], DON can reduce the villus height and the number of goblet cells but increased crypt depth. Intestinal tight junctions (TJs) play a crucial role in maintaining the intestinal epithelial cell barrier and intestine health; the expression of TJ proteins (TJPs) down-regulated under the effect of DON can cause impairment of the intestinal barrier and an increase in intestinal permeability [18,19]. Furthermore, DON can activate the related proinflammation signaling pathways such as ERK-p38, NF-κB and MAPK pathways to up-regulate the expressions of inflammatory factors (IL-6, TNF-α, IL-1β, etc.), thus promoting the occurrence of inflammation [13,20,21]. In addition, the gut microbiota, considered an important indicator of host health associated with a variety of diseases, can be destroyed with DON exposure, leading to changes in the richness (Chao1) and evenness index (Shannon), changes to the major bacterial flora at the phylum or genus level, an increase in the proportion of harmful bacteria and a decrease in the proportion of probiotics [22,23]. Gut microbiota disruption may directly affect the normal operation of intestinal functions, and the occurrence of some intestinal disease may be related to it [17].

As has been stated, DON can induce intestinal injury in vitro and in vivo through different aspects and different levels of damage. Previous research has focused on intestinal barrier damage, intestinal inflammation and gut flora but has not yet established the intrinsic interactions of the injury mechanism. Thus, in the present study, we conducted a comprehensive study on the toxicity of DON from detection to various toxic mechanisms in vivo and in vitro. DON exposure in an in vitro model in IPEC−J2 cells and an in vivo model in Kunming mice was established. Furthermore, the intrinsic interactions from multiple aspects, including intestinal structure, intestinal barrier, gut microbiota and intestinal inflammation, on intestinal toxicity of DON were deeply analyzed through molecular biology, cytology and animal experiments. The results will provide ideas for exploring novel prospective approaches to alleviate the DON toxicity.

## 2. Results

### 2.1. DON Contamination at Harvest in Shaanxi

**Figure 1:**
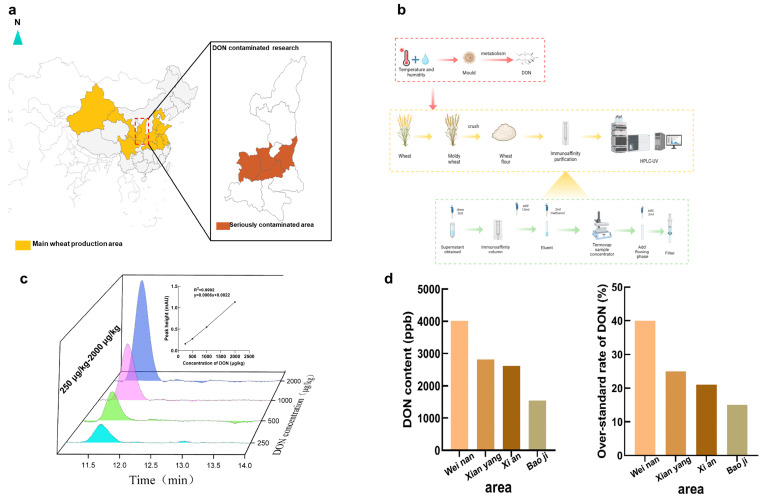
Investigation on DON contamination in the main wheat production areas of Shaanxi. (**a**) The main contamination distribution of DON in Shaanxi; (**b**) detection process of DON in wheat; (**c**) DON standard curve; (**d**) DON contamination rate.

A survey was conducted on the content of DON concentration in wheat at harvest in 2023 from Shaanxi Province, a prominent wheat production region in China (Figure 1a). The primary focus of our collection efforts was on wheat sourced from the central regions, known for having the highest wheat output in Shaanxi Province. As shown in Figure 1b, the collected wheat was ground into flour, purified, centrifuged to remove the supernatant and, finally, filtered and the DON content in wheat was detected by high-performance liquid chromatography. The standard curve generated by the DON standard test of different concentrations is used to detect the DON content in the sample (Figure 1c). The results revealed a severe DON contamination in wheat, especially in the central area of Shaanxi, the highest pollution concentration could reach 4 mg/kg, four times higher than the limitation standards of DON in China. Additionally, another dataset indicated that 40% of the wheat samples in this region were above the established threshold for DON levels. This result showed the severe DON pollution in wheat during the harvest time in Shaanxi; it may be attributed to the continuous raining during the wheat harvest season in May. Thus, at a suitable temperature (30–35 °C) and humidity condition, with a naturally nutritious wheat culture medium, the parasitic Fusarium could thrive and generate significant quantities of DON.

### 2.2. Toxicity of DON in IPEC−J2

**Figure 2:**
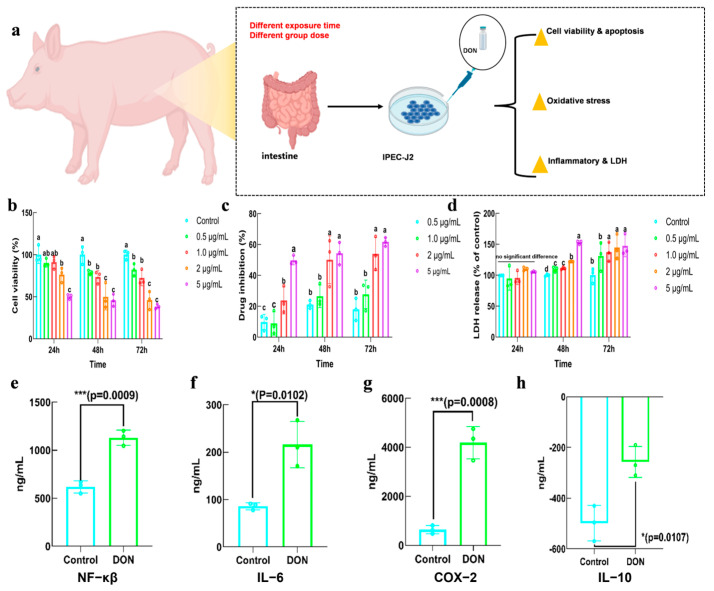
Effects of DON on IPEC−J2 cell viability, LDH release and inflammation. (**a**) DON IPEC−J2 cytotoxicity assay flow; (**b**) effect of DON on cell viability at 24, 48 and 72 h; (**c**) effect of DON on drug inhibition at 24, 48 and 72 h; (**d**) effect of DON on LDH release at 24, 48 and 72 h; (**e**) the effect of DON on NF-κβ expression; (**f**–**h**) the effect of DON on inflammation factors of IL-6, COX-2 and IL-10. Bars marked with different lowercase letters were significantly different (*p* < 0.05). Bars marked with * and *** were significantly different with *p* < 0.05 and *p* < 0.001, respectively. Notes: the negative co-ordinates in Figure h represent the negative feedback relationship between IL-10 and induced inflammation.

IPEC−J2 cell line was utilized to investigate the intestinal damage induced by DON. The detrimental effects of DON exposure on IPEC−J2 cells were evaluated by measuring cell viability, antioxidant indicators, LDH release, inflammatory markers and cell apoptosis (Figure 2a). Figure 2b showed the impact of various dosages of DON on cell viability at 24, 48 and 72 h, respectively. It demonstrated that cell viability declined in a way that was dependent on both time and dosage of DON. Notably, there was a significant decrease in cell viability as the concentration of DON increased to 2.0 μg/mL. In terms of time, cell viability decreased considerably during 24–48 h of DON exposure, while it showed no significant decrease during 48–72 h. The drug inhibition rate exhibited a comparable outcome to the cell viability, as seen in Figure 2c. The results of Figure 2d indicated that DON had no significant effect on the LDH release of IPEC−J2 in 24 h, but LDH release was increased significantly at 2.0 mL of DON in 48 h and 72 h. Interestingly, the indicators of cell viability and LDH release showed that the effect of DON on cells changed insignificantly after the concentration increased from 2.0 to 5.0 μg/mL; 2.0 μg/mL was thus selected as the exposure dose in subsequent experiments. The effect of DON exposure on the inflammatory level of IPEC−J2 was also detected, and the results showed that NF-κβ, a key regulator of inflammation, was activated. Additionally, the expression of IL-6 and COX-2 were increased, whereas IL-10 was decreased (Figure 2e–h).

**Figure 3:**
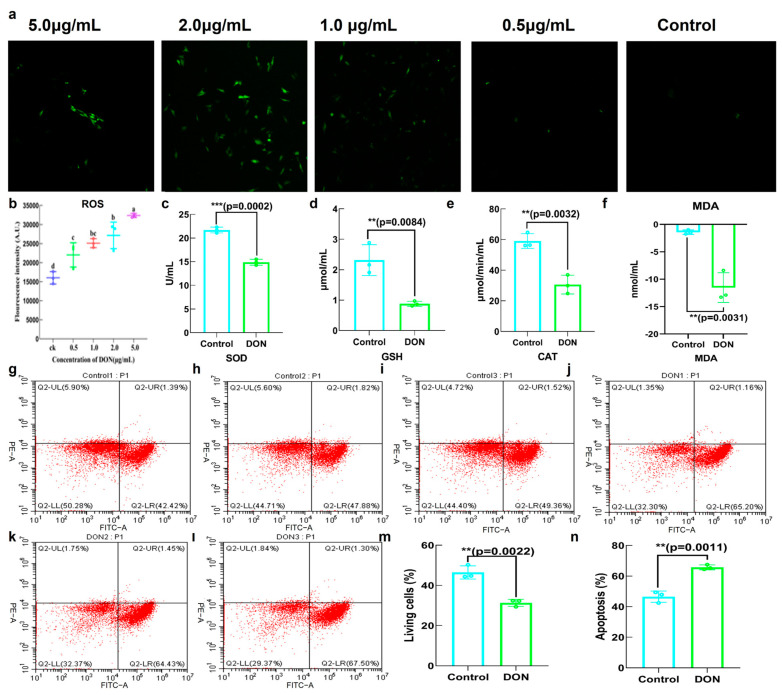
Effects of DON on IPEC−J2 oxidative stress and cell apoptosis. (**a**,**b**) Effect of DON on level of ROS; (**c**–**f**) effect of DON on SOD, GSH, CAT and MDA content; (**g**–**l**) the cell apoptosis of IPEC−J2 after DON exposure; (**m**,**n**) comparison of living cell ratio and apoptosis ratio between control group and DON group. Bars marked with ** and *** were significantly different with *p* < 0.01 and *p* < 0.001, respectively. Bars marked with different lowercase letters were significantly different (*p* < 0.05). Notes: the negative co-ordinates in Figure (**f**) represent the negative feedback relationship between MDA and antioxidant.

Through the analysis of relative fluorescence intensity, it was observed that the presence of DON resulted in an augmentation of reactive oxygen species (ROS) levels. Additionally, there was a significant enhancement in fluorescence intensity with the increase in DON concentration, especially at concentrations of 2.0 and 5.0 μg/mL (Figure 3a,b). In addition, various indicators associated with antioxidants demonstrated a decrease in the levels of SOD, GSH and CAT, whereas the level of MDA exhibited an increase (Figure 3c–f). According to the analysis of cell apoptosis, the proportion of living cells was down-regulated and the proportion of apoptosis was up-regulated after exposure to DON (Figure 3g–n).

### 2.3. The Effects of DON on General Physical Indicators in Mice

**Figure 4:**
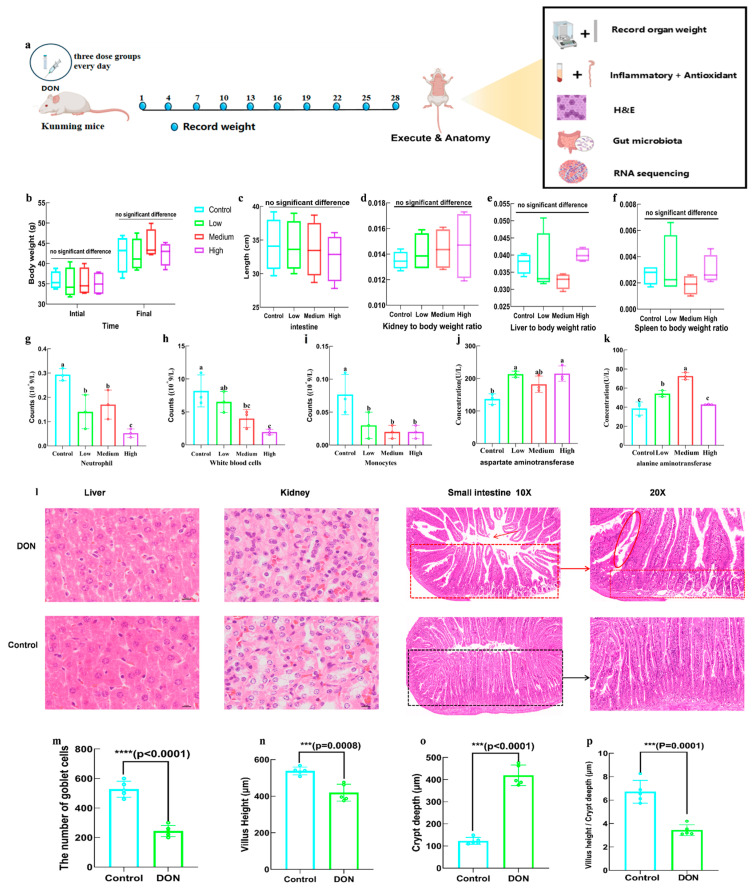
Effects of DON on basic indicators of Kunming mice. (**a**) DON animal toxicity experimental process; (**b**) effect of DON on initial and final body weights; (**c**) effect of DON on small intestine length; (**d**–**f**) effects of DON on the body weight ratio of kidney, liver and spleen, respectively; (**g**) effects of DON on counts of neutrophil; (**h**) effects of DON on counts of white blood cells; (**i**) effects of DON on counts of monocytes; (**j**) effects of DON on concentration of aspartate aminotransferase; (**k**) effects of DON on concentration of alanine aminotransferase. (**l**) The histopathology of liver, kidney and intestinal tissues, with magnification 10× and 20×; (**m**) effect of DON on the number of goblet cells; (**n**) effect of DON on villus height; (**o**) effect of DON on crypt depth; (**p**) effect of DON on villus height/crypt depth. Bars marked with *** and **** were significantly different with *p* < 0.001 and *p* < 0.0001, respectively. Bars marked with different lowercase letters were significantly different (*p* < 0.05). Notes: the box and line segments in Figure (**a**) indicate that the area is magnified in the following figure.

The effects of DON on several physical indicators, including weight indicators, hematological analysis, serum analysis, relative organ weight measures and H&E staining, are presented in Figure 4. The results indicated that there was no notable difference in body weights between various doses of DON exposure and the control group. The weights of the organs, such as liver, heart, kidney and spleen, in all mice were measured and the relative weights of the organs were determined. Figure 4d–f indicated that there were no significant changes in the relative weight of the heart and kidneys. However, exposure to a high dosage of DON could result in a significant increase in the relative weights of liver and a decrease for spleen. The white blood cell, monocyte and neutrophil counts in the DON groups significantly decreased, whereas the concentration of aspartate aminotransferase and alanine aminotransferase exhibited a notable rise (Figure 4g–k). Additionally, the histology of liver, kidney and intestine tissues were observed under a 20 × microscope; the results indicated that, in liver and kidney, DON exposure groups had intact organs without damage or injury and the organs were neatly ordered. However, the small intestine in DON exposure groups exhibited anomalies compared with the normal group (Figure 4l); it had a decrease in the number of goblet cells, the height of the villus and the ratio of villus height to crypt depth but an increase in the crypt depth (Figure 4m–p).

### 2.4. The Effects of DON on Intestinal Metabolic Pathways and Gene Expression

**Figure 5:**
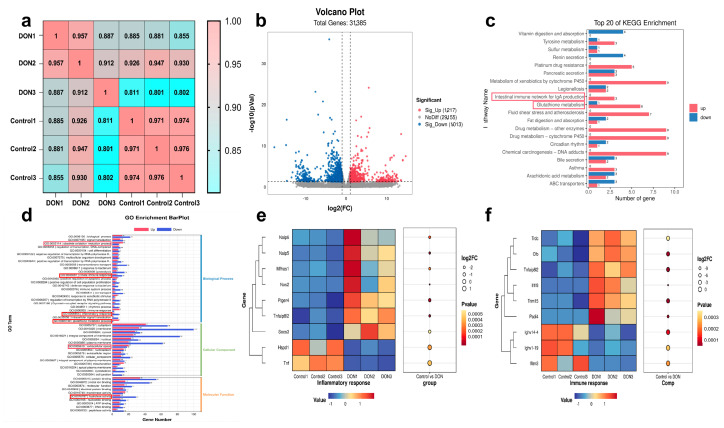
Transcriptomic analysis with DON exposure. (**a**) The intra-group and extra-group correlations among samples; (**b**) volcano plot of differentially expressed genes; (**c**) KEGG pathway enrichment analysis; (**d**) GO enrichment plot analysis; (**e**) the different expressed genes related to inflammation response; (**f**) the different expressed genes related to immune response. The different expressed genes related to immune response. Notes: the red boxes indicate several related pathways involved in the article.

To further investigate the effect of DON exposure in mice, small intestine RNA sequencing was conducted. Figure 5a demonstrates that the correlation between samples within the group was greater than that of samples between the groups. The volcano plot in Figure 5b reveals that a total of 2230 genes of different expression were detected, with 1217 genes up-regulated and 1013 genes down-regulated. The KEGG analysis identified 20 pathways that exhibited substantial differences (Figure 5c); during these pathways, drug metabolism, glutathione metabolism, metabolism of xenobiotics via cytochrome P450 and gut immune network for lgA synthesis were the most prominently represented pathways. Based on the GO analysis in Figure 5d, the genes involved in the obsolete oxidation−reduction process, immune and inflammatory response and glutathione metabolism process were significantly expressed in the biological process. In terms of cellular components, significantly expressed genes were mainly concentrated in the extracellular space, plasma membrane, extracellular area and cell junction. And genes involved in protein binding, transferase activity, hydrolase activity, ATP binding and DNA binding were mainly expressed in terms of molecular function. In addition, gene expression involved in immune and inflammatory responses was also analyzed. Figure 5e,f show that DON exposure exhibited a significant up-regulation of the genes Naip6, Naip5, Mfhas1, Nos2, Ptger4, Tnfaip812 and Socs3 and a down-regulation of the genes Hspd1 and Tnf in the inflammatory response pathway. In the immune response pathway, DON exposure increased the expression of the Trdc, Cfb, Tnfaip812, Il1f8, Trim15 and Padi4 genes, while it decreased the expression of the lghv14−4, lghv1−19 and Ifitm3 genes.

### 2.5. The Effects of DON on Gut Microbiota

**Figure 6:**
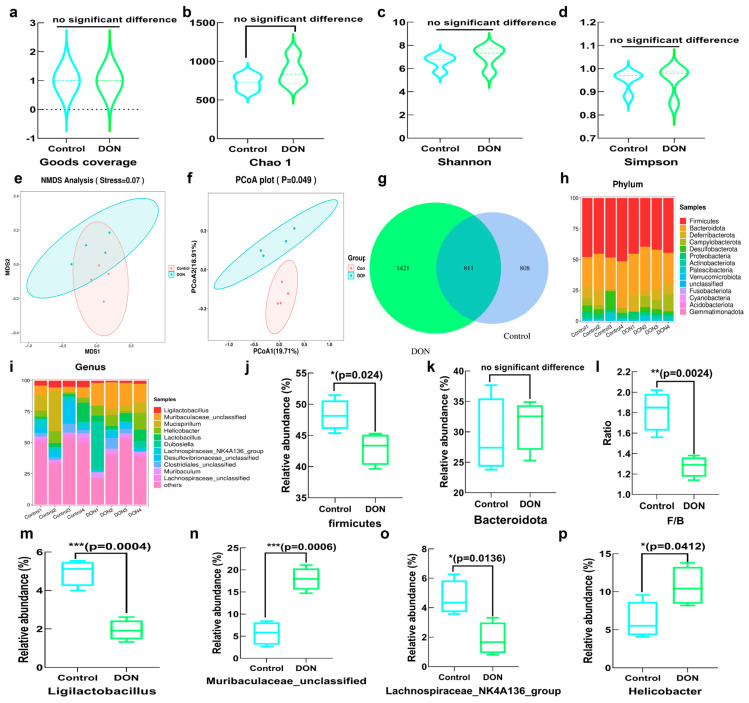
Gut microbiota analysis with DON exposure. (**a**) Goods coverage plot; (**b**) observed otus plot; (**c**) Chao 1 plot; (**d**) Shannon plot. (**e**) NMDS analysis; (**f**) PCOA score plot of the features; (**g**) Venn diagram; (**h**) gut microbial composition at the phylum level; (**i**) gut microbial composition at the genus level; (**j**) effect of DON on relative abundance of *firmicutes*; (**k**) effect of DON on relative abundance of *Bacteroidates*; (**l**) effect of DON on ratio of *Firmicutes*/*Bacteroidetes* (F/B); (**m**) effect of DON on relative abundance of *Ligilactobacillus*; (**n**) effect of DON on relative abundance of *Muribaculaceae*; (**o**) effect of DON on relative abundance of *Lachnospiraceae_NK4A136_group*; (**p**) effect of DON on relative abundance of *Helicobacter*. Bars marked with *, ** and *** were significantly different with *p* < 0.05, *p* < 0.01 and *p* < 0.001, respectively.

The fresh feces of mice were collected for 16S rDNA sequencing. The number of distinct species and their variety within various groups was conducted; the coverage of each sample was uniformly 1, which indicated that the sequencing results were considered genuine and reliable (Figure 6a). There was no statistically significant difference between DON exposure and the control group in Chao, Simpson and Shannon indexes, which showed no difference in species diversity (Figure 6b–d). The PCOA and NMDS plot both indicated a substantial distinction between the gut microbiota of the control group and the DON exposure group in Figure 6e,f. The Venn diagram illustrated a total of 811 overlapping ASVs between the two groups. DON exposure group owned 1421 unique ASVs, while the control group was 808 (Figure 6g). Additionally, the relative abundance of the dominant flora at both the genus and phylum levels is displayed in Figure 6h,i. At the phylum level, the DON exposure group not only decreased the relative abundance of firmicutes but also decreased the F/B ratio (Figure 6j–l). At the genus level, there was a significant decrease in the relative abundance of *Ligilactobacillus* and *Lachnospiraceae_NK4A136_group*, whereas there was a significant increase in the relative abundance of Helicobacter and *Muribaculaceae* after DON exposure (Figure 6j–p).

**Figure 7:**
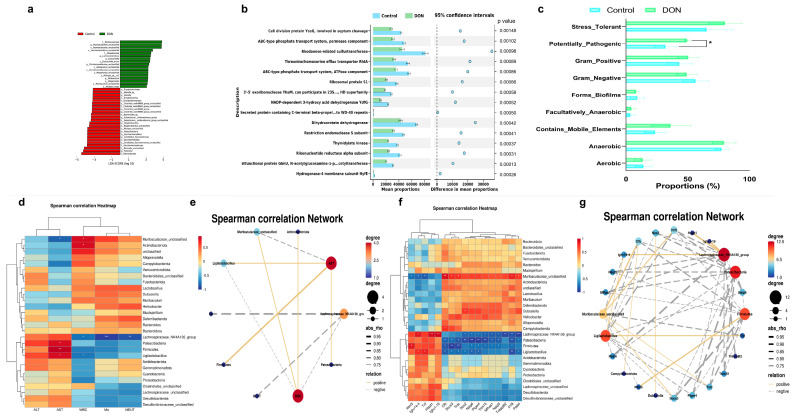
Correlation analysis between gut microbiota and other indicators. (**a**) Biomarker analysis by LefSe; (**b**) functional analysis of gut microbiota prediction; (**c**) predictive analysis of gut microbiota phenotypes; (**d**) correlation analysis heatmap between gut microbiota and serum indicators; (**e**) correlation analysis network between gut microbiota and serum indicator; (**f**) correlation analysis heatmap between gut microbiota and genes; (**g**) correlation analysis network between gut microbiota and genes. Bars marked with *, ** and *** were significantly different with *p* < 0.05, *p* < 0.01 and *p* < 0.001, respectively.

In order to conduct a more comprehensive investigation on the harmful impact of DON on gut microbiota, LEfSe analysis was employed to identify the biomarker in the presence and absence of DON exposure. The analysis indicated that *Muribaculaceae*, *Lachnoclostridium*, *Duncaniella*, *Christensenellaceae_unclassfied*, *Allobaculum*, *Megamonas* and *Atopobiaceae* were enriched at the phylum and genus level after DON exposure (Figure 7a). The predicted function of gut microbiota was analyzed by PIRUST2 analysis with Clusters of Orthologous Groups (COG); it revealed that the predominant functions of the gut microbiota such as ribosomal protein, permease component, some transport systems, rhodanese-related sulfurtransferase, some dehydrogenases, thymldylate kinase, etc., were impaired after DON exposure, also accompanied by the increasing of the relative abundance of potentially pathogenic bacteria (Figure 7b,c).

Spearman analysis was used to investigate the association between gut microbiota and blood serum indicators/key genes (Figure 7d,e); the presence of *Muribaculaceae* and *Actinobacteriota* showed a positive correlation with white blood cell count (WBC), whereas *Ligilactobacillus* and *Lachnospiraceae_NK4A136_group* showed a negative correlation with it. Additionally, *Lachnospiraceae_NK4A136_group* showed a negative correlation with monocytes (Mo) and neutrophils (NEUT). Furthermore, there was a negative correlation between *Muribaculaceae* and AST, while *Patescibacteria*, *Firmciutes* and *Ligilactobacillus* showed a positive correlation with AST. Figure 7f,g demonstrate a positive correlation between *Muribaculaceae* and Padi4, Il1f8, Trdc, Socs3 and Cfb and a negative correlation with Tnf, lghv14−4 and Ifitm3. The presence of *Lachnospiraceae_NK4A136_group* showed a positive correlation with lghv1−19, Hspd1 and lghv14−4 but a negative correlation with Tnfaip812, Il1f8, Trim15, Padi4, Naip5, Mfhas1, Ptger4, Naip6 and Nos2. *Patescibacteria* had a negative correlation with Il1f8, Trim15, Padi4, Naip5, Mfhas1, Ptger4, Naip6, Nos2, Trdc, Socs3 and Cfb. *Firmicutes* had a negative correlation with Il1f8, Trim15, Naip5, Mfhas1, Ptger4, Naip6, Trdc, Socs3 and Cfb but showed a positive correlation with Ifitm3. *Ligilactobacillus* had a negative correlation with Il1f8, Padi4, Ptger4, Naip6, Nos2, Trdc, Socs3 and Cfb, while showing a positive correlation with Tnf and lghv14−4.

## 3. Discussion

The fluctuating climatic conditions during wheat harvest might result in the proliferation of mold and the generation of DON. The prevalence of deoxynivalenol (DON) contamination in key wheat-producing regions due to unpredictable and volatile climatic conditions has remained a significant concern [1]. Although the weather effect in 2023 was unintentional, it also demonstrated that DON was susceptible to contamination during the growing and storage of grains, which should be a long-term concern. The toxicity of DON not only affects animals but also humans; long-term chronic direct or indirect exposure to DON can also cause similar damage [5]. Hence, it is crucial to investigate the effect of DON-induced harm and the different forms of damage in the gastrointestinal system.

LDH release serves as a reliable indicator, as a constant cytoplasmic enzyme present in all cells. LDH release into the cell culture supernatant occurs swiftly upon the occurrence of damage to the plasma membrane, representing a vital characteristic observed in cells when experiencing cellular injury [24,25]. ROS levels represented that cellular oxidative stress not only led to damage in cell membranes and organelles but also potentially caused mitochondrial DNA damage and further exacerbated oxidative stress [26,27]. The results in this study demonstrated that ROS levels were increased due to DON exposure, which could trigger the production of inflammatory factors such as TNF-α, IL-1β, IL-6, etc., thus causing cell damage and even programmed cell death/apoptosis [28]. The proinflammatory factor IL-6 and COX-2 were studied in this study and consequently increased after DON exposure, accompanied by a decreased level of anti-inflammatory factor IL-10. Exposing HIEC-6 cells to DON for 24 h could obtain a similar result of significant oxidative stress and an inflammatory response, characterized by increased levels of IL-6 [29]. This suggests a strong correlation between exposure to DON and levels of reactive oxygen species (ROS) and inflammation in IPEC−J2 cells. 

In addition to causing ROS and inflammatory responses in IPEC−J2 cells, a chronic DON exposure can also cause damage to various toxic responses in the body, including body weight decreasing, inflammation emergency, blood indicators changing and substantial organ destruction and functional damage [3,30]. Although there were no significant differences observed in body weights in the present investigation, the levels of white blood cells, monocytes and neutrophils decreased significantly after DON exposure, representing that the immune system was weakened by exposure to DON, which may cause a lack of immunity. Due to the inflammation being one of the main symptoms of many diseases and that it could be reflected through serum and hematology indicators, the activities of alanine aminotransferase (ALT) and aspartate aminotransferase (AST) in serum were analyzed and the results revealed a significant increase after DON exposure, which represented valuable indicators of hepatic injury and dysfunction, providing evidence of organ injury by DON [31,32]. What is more, a notable disruption in the morphology of the small intestine was caused by DON exposure, including a decrease in the number of goblet cells, the height of the villus and the ratio of villus height to crypt depth but an increase in the depth of the crypt, which are strongly correlated with the digesting capacity, thus having a negative impact on the regular function of the intestine [4,33]. In addition, DON exposure can also significantly change substance metabolism, nutrient absorption, immune capacity and inflammatory response of the intestine [34,35,36]. RNA sequencing reflected that toxicity effects of DON on mice remained significant at the genetic level. After DON exposure, genes were significantly enriched in the immune pathway, glutathione pathway and inflammatory pathway, which aligned with the antioxidant and anti-inflammatory factors analyzed in this study, confirming the toxic effect of DON on the antioxidant and anti-inflammatory capacities in the body.

Gut microbiota act as a connection between the phenotype of the host and the functional activities of tissues/organs and has received more and more attention in either nutritional or toxicity research [37]. It is still uncertain whether gut microbiota has a role in mediating immunological diseases and intestinal damage caused by DON. The results in this study revealed that the balance of gut microbiota could be destroyed with chronic DON exposure by decreasing beneficial and increasing harmful flora. With *Firmicutes* (F) and *Bacteroidetes* (B) being two dominant phyla representing together up to 90% of the total gut microbiota, the *Firmicutes*/*Bacteroidetes* (F/B) ratio once had been suggested as an important index in gut microbiota health [38,39] and was decreased with DON exposure, accompanied by the decrease in firmicutes relative abundance, which is consistent with most studies [4,34]. At the genus level, DON exposure significantly up-regulated the relative abundance of *Helicobacter pylori*, a type of *Helicobacter*, which was once considered to be associated positively with proinflammatory cytokines and varieties of diseases and was up-regulated to disrupt flora balance and promote intestinal inflammation [40,41]. In contrast, the relative abundances of *Ligilactobacillus*, *Lachnospiraceae_NK4A136_group* and *Muribaculaceae* were all down-regulated after DON exposure, with *Ligilactobacillus* being a probiotic showing potential in preventing various diseases such as treating obesity, alleviating constipation and resisting sepsis-associated acute liver injury [42,43,44]. Meanwhile, for *Lachnospiraceae_NK4A136_group*, a potential anti-inflammatory flora associated with some drugs for obesity and depression treatment, its vital role of anti-inflammatory effect was also weakened by DON [45,46,47]. Interestingly, although some studies indicated that *Muribaculaceae* has a positive correlation with food allergy, it was still considered to be healthy for the gut microbiota in many aspects [48,49]; the down-regulation of *Muribaculaceae* in this study may be related to the active protection and compensatory protection mechanism of intestinal flora [30,50]. Compared with other studies, DON exposure destroyed the balance of intestinal flora and reduced the abundance of firmicutes, the main flora, but there were also differences in some specific bacterial composition changes, which were mainly caused by the difference in the dose of DON, exposure time and subjects [51]. Moreover, with Lefse analysis, DON exposure biomarkers were discovered, the functions of gut microbiota and the proportions of various bacteria were predicted. It indicated that DON exposure may bring about weakening of the hydrolysis and transport functions related to the flora, and increasing the proportion of potential pathogenic bacteria was analyzed, which may help provide some characteristic physiological/biochemical indicators for the later treatment of DON. Furthermore, a significant interaction relationship between the gut microbiota and the multiple serum indicators/genes was discovered in our further correlation analysis, showing systemic complexly toxic effects in the whole body, with the most significant being in intestinal injury [3]. As previously mentioned, DON exposure seriously destroyed the balance of gut microbiota, cause intestinal injury and even affect general health. Therefore, it is crucial for nutritionists to consider the potential approaches for enhancing the gut microbiota in order to mitigate the harmful consequences of prolonged exposure to DON in the human body. 

## 4. Conclusions

The issue of DON contamination continues to be prevalent globally, presenting a significant risk to both food safety and human health. The effects of DON on intestinal toxicity were multifaceted, including weakening the intestinal barrier, inducing inflammation, disordering the immune system and destroying the gut microbiota. These various levels of damage interacted with each other, with a particularly strong association observed between the main gut flora and specific markers of toxicity. Nonetheless, it is worth noting that certain polyphenols, bioactive proteins and some essential nutrients such as SeNPs with antioxidant capacity have been reported to shield people from the harmful effects of DON by elucidating and clarifying the mechanisms of damage. They could alleviate DON toxicities through modulating different signaling pathways and/or gut microbiota. This research is expected to provide better insights and research directions for alleviating DON injury through a comprehensive study on the toxicity of DON.

## 5. Materials and Methods

### 5.1. Chemicals and Reagents

IPEC−J2 cells were purchased from Icell bioscience company (Shanghai, China). The Kun Ming (KM) mice were purchased from Xi’an Jiaotong University Animal Center (Xi’an, China). Deoxynivalenol (DON) and immunoaffinity columns of DON were purchased from Pribolab company (Qingdao, Shandong, China). Fetal bovine serum (FBS), antibiotic and 1640 medium were purchased from Siji chun company (Shaoxing, Zhejiang, China) and Procell company (Wuhan, China). ROS assay kits, CCK−8 assay kits, ELISA assay kits and Annexin V−FITC/PI assay kits were purchased from Solarbio company (Beijing, China), Beyotime Biotechnology company (Nantong, China), Jianglaibio company (Shanghai, China) and Nanjing Jiancheng biology company (Nanjing, China), respectively. Other reagents used in this study were all purchased from a local reagent company.

### 5.2. Wheat Sample Collection and DON Detection

Four wheat samples collected from Xi’an, Baoji, Xianyang and Weinan in Shaanxi Province were pulverized into flour. Then, 5 grams of the powdered wheat were dissolved in 20 milliliters of distilled water. Subsequently, they were placed in a shaker at 200 rpm for 20 min. After that, they were centrifuged at 4000 rpm for 5 min. Subsequently, 2 mL of supernatant was added into the immunoaffinity column to conduct the DON extraction process with 2 mL of methanol. Finally, the dehydrated extract was obtained after nitrogen evaporation. The dehydrated extract was redissolved with 2 mL 20% methanol and filtered through 0.22 μm filter membrane. The liquid that was passed through a filter was identified using high-performance liquid chromatography. HPLC/UV System with C18 column (4.6 × 250 nm, 5 mm) was used to analyze DON under the detection process of isocratic elution with a methanol/water 20:80 (*v*/*v*) mobile phase at 35 °C and 10 μL of injection volume with a 0.8 mL/min flow rate under 218 nm wavelength.

### 5.3. In Vitro Cytotoxicity of DON

#### 5.3.1. The Effect of DON on Cell Viability and Drug Inhibition Rate

IPEC−J2 cells were cultured in ordinary 1640 medium supplemented with 10% fetal bovine serum (FBS) and 1% antibiotics, placed in an incubator with 5% CO_2_ at 37 °C. The viability of IPEC−J2 cells and effect of DON on IPEC−J2 cell inhibition rate were detected with Cell Counting Kit−8 (CCK−8) following the manufacturer’s instruction. Cells were initially placed in a 96-well plate, and DON was added when the cells reached 70% confluence. Then, 10 μL of CCK-8 solution was added to each well after DON exposure for 24, 48 and 72 h, respectively.

#### 5.3.2. The Effect of DON on Lactate Dehydrogenase (LDH) Release

The LDH release was detected using a commercial LDH assay kit (Beyotime, Shanghai, China), and cells were cultured in the same way as the treatment of cell proliferation analysis. Afterward, the 96-well microplate was centrifugated at 400× *g* for 5 min and the supernatant was carefully removed and transferred into a new 96-well microplate. Subsequently, 60 µL of the LDH working solution was added to the microplate and incubated for 30 min at room temperature, while ensuring it remained shielded from light.

#### 5.3.3. The Effect of DON on Cell Apoptosis

IPEC−J2 cells were assigned to the control and DON exposure group. The DON group was exposed to 2.0 μg/mL DON solution for 48 h when the cells were about 80–90% confluence. Then, the Annexin V−FITC/PI Apoptosis Detection Kit was used to detect the cell apoptosis by analytical flow cytometry (Beckman, Suzhou, China).

#### 5.3.4. The Effect of DON on Inflammatory Factor Expression

IPEC−J2 cell culture was centrifuged at 2000× *g* for 20 min at 4 °C and the supernatant was collected for use. The levels of inflammatory factors IL-6, COX-2, IL-1 and IL-10 were measured by ELISA kits from Jianglai Biology (Shanghai, China).

#### 5.3.5. The Effect of DON on Antioxidant Capacity of IPEC−J2

IPEC−J2 cells were separated into four groups with DON and distilled water for 48 h. The observation of reactive oxygen species (ROS) was conducted using a High-Resolution Laser Confocal Microscope (Laika, Wezla, Germany) with an excitation wavelength of 488 nm and an emission wavelength of 525 nm. Other antioxidant indicators such as SOD, CAT, GSH and MDA were detected by chemical kits, and the value was recorded using the fluorescent microplate reader.

### 5.4. In Vivo Cytotoxicity of DON

#### 5.4.1. Animals Feed and Observation

The animal study complied with all institutional and national guidelines and was approved by Shaanxi Normal University Animal Ethics Committee. The six-week-old Kunming rats were observed for a week without disease and were separated into the control group and three doses of DON groups (n = 8/dose group, all male) with 2 mg/kg, 0.2 mg/kg and 0.02 mg/kg by intragastric administration once daily. Accordingly, the control group was administered equal volumes of tap water without DON. Mice were maintained on a 12 h light/dark cycle and received a commercial standard mouse cube diet with tap water ad libitum in accordance with the NIH Guide for the Care and Use of Laboratory Animals. The body weight of the mice was recorded every three days. After 30 days of administration, mice were all killed after 8 h of restricted food. Finally, blood was collected from mice under general anesthesia and was centrifuged at 3000× *g* to obtain serum. Liver, kidney and small intestine tissues were fixed with 4% paraformaldehyde at 4 °C, and remaining tissues were preserved at −80 °C for subsequent analysis. For H&E staining, fixed tissues were made into paraffin sections, stained with hematoxylin and eosin and a DP72 digital microscope camera (Olympus, Tokyo, Japan) was used to photograph sections.

#### 5.4.2. RNA Sequencing Analysis

Total RNA was extracted from mice’s gut by Trizol reagent (thermofisher, 15596018). After that, mRNA was purified from total RNA (5 μg) using Dynabeads Oligo (dT) (Thermo Fisher, California, CA, USA) with two rounds of purification. Then, mRNA was fragmented into short fragments using divalent cations under elevated temperature (Magnesium RNA Fragmentation Module under 94 °C for 5–7 min). The total RNA quantity and purity were analyzed by Bioanalyzer 2100 and RNA 6000 Nano Lab Chip Kit (Agilent, CA, USA, 5067−1511); high-quality RNA samples with RIN number > 7.0 were used to construct a sequencing library. RNA sequencing was conducted by LC−BIO Technologies Co., Ltd. (Hangzhou, China). We utilized GO and KEGG gene databases to specifically analyze the data obtained by RNA sequencing, screened out the pathways with significant gene enrichment changes in each database and further discussed the changes in genes in inflammatory stress and immune stress pathways closely related to the influence of DON, so as to further explore the influence of DON at the gene level of mice.

#### 5.4.3. Gut Microbiota Analysis

Fresh feces of mice receiving the highest were collected before the end of the experiment and the genomic DNA was extracted from fresh feces using the CTAB. Lc−Bio Technology Co., Ltd. (Hangzhou, China) conducted the complete procedures of 16S rDNA sequencing and analysis. The V3−V4 region of 16S rDNA was amplified with the primers of 341F (5′−CCTACGGGNGGGCWGCAG−3′) and 805R (5′−GACTACHVGGGTATCTAATCC−3′). The 5′ ends of the primers were tagged with specific bar codes per sample and sequencing universal primers. Samples were sequenced on an Illumina NovaSeq platform according to the manufacturer’s recommendations, provided by LC−Bio. Data profiling was carried out on the basis of the above-described methods by DADA2, Vsearch software (v2.3.4), fqtrim (v0.94), SILVA (release 138), QIIME2, Alpha diversity analysis, etc., and the graphs were drawn by R 4.1.3 package.

### 5.5. Statistical Analysis

The experiments were generally performed in triplicate, and the data are presented as means ± standard deviations. All data were subjected to one-way analysis of variance with SPSS 27 software (SPSS Institute Inc., Cary, NC, USA). The data were considered statistically significant when *p* < 0.05.

## Data Availability

The data presented in this study are available on request from the corresponding authors.
